# Analysis of Physical and Mechanical Properties of Universal Composites under Different Types of Polishing before and after Acid Challenge

**DOI:** 10.1155/2024/6817593

**Published:** 2024-02-29

**Authors:** José Roberto Vergínio de Matos, Letícia Barbero Antunes, Isabela Araguê Catanoze, Isabela Saturnino de Souza, Paulo Henrique dos Santos, Aimée Maria Guiotti

**Affiliations:** ^1^Department of Dental Materials and Prosthodontics, School of Dentistry, São Paulo State University (UNESP), Araçatuba, São Paulo, Brazil; ^2^Dental Research Institute, Restorative Dentistry, Faculty of Dentistry, University of Toronto, Toronto, Canada

## Abstract

This study aimed to evaluate in vitro the degree of surface smoothness provided by two different polishing techniques and the effect of acid challenge on the alteration of surface roughness (Ra), microhardness (Knoop), and color (*Δ*E_00_) of three nanoparticulate composites, simulating 1 year of exposure to hydrochloric acid (HCl). Eighty specimens for each composite were divided into four groups (*n* = 240), being control without polishing, control with wear, WPC (wear + polishing with Cosmedent Kit), and WPB (wear + BisCover LV liquid polish). Repeated measures ANOVA was applied for Ra and Knoop Microhardness. For the color (*Δ*E) three-way ANOVA was applied. In cases of statistically significant the Tukey posttest was applied (*α* = 0.05). Both types of polishing tested resulted in a surface smoothness below the critical value established by the studies (Ra ≥ 0.2 *μ*m), even after immersion. The microhardness of all composite resins decreased after the challenges. The specimens immersed in HCl showed a lower microhardness (42.2 Kgf/mm^2^) when compared to the specimens immersed in artificial saliva (44.7 Kgf/mm^2^). Regarding the color change, the composites presented values compatible with clinical acceptability, with a statistically significant difference only between the control group and the other types of polishing for the Z350 XT resin (*Δ*E_00_ = 3.78). It was concluded that both mechanical and chemical polishing produced a satisfactory surface smoothness, even after immersions in artificial saliva and HCl. The microhardness of the composites was affected by the challenges and the composites tested were within clinical acceptability with regard to color change.

## 1. Introduction

The loss of dental hard tissue by a noncarious process is defined as erosion, caused by intrinsic or extrinsic acids [1–3]. Intrinsically, erosion is generated by the presence of gastric juice in the oral cavity, composed mainly of hydrochloric acid. This happens when the patient has a bulimia nervosa condition, where he self-induces vomiting, or in the presence of gastroesophageal reflux disease [2, 4, 5].

As an extrinsic factor, there is the ingestion of some beverages such as soft drinks, juices, coffee, and wines. As well as the tooth structure, the restorative materials are also subject to the action of acids, which can degrade the matrix and the load of the material, due to the acid pH and because they contain pigments, inducing color changes, loss of polish, wear, and increased surface roughness, which consequently reduces the life of restorations and prostheses [6–10]. Regarding the material to be used in noncarious cervical lesions, it has been stated that composite resin restorations are a general indication based on their good esthetic properties and clinical performance [11] and such restorations are an appropriate method for preventing further deterioration [12]. A systematic review and meta-analysis indicated glass ionomers had better retention than composite resin but inferior surface roughness and color matching [13]. Thus, composite resin with nanosized particles (nanocomposites) become a great material option, as they combine resistance and excellent polishing, minimizing biofilm adhesion, and gingival inflammation generated by the roughness of materials such as glass ionomer.

The growing search for better aesthetic results in the dentistry has driven the development of new resin materials in order to improve their mechanical properties, optics, and clinical application [14, 15]. Modifications to these composites have been proposed, as the incorporation of new monomers and initiators and the application of new technologies in its manufacture [16]. One of the most significant advances in recent years has been the use of nanotechnology through the incorporation of nanosized particles into the resin matrix, emerging a new class of resins, and the nanocomposites [17–21]. The main claimed advantages of nanocomposites over other composite materials include a high surface/volume ratio that allows small filler size and reduced interparticle separation, enhanced mechanical properties, high ductility without strength loss, scratch resistance, improved optical properties (light transmission depends on particle size), and improved thermal properties [22, 23]. Especially for dental nanocomposites simplified and enhanced aesthetic properties such as high gloss and gloss stability and excellent polishability and adaptability are claimed by their manufacturers.

Nanotechnology has provided the improvement of composite resin with the promise of improving the properties of these materials, making it possible to perform better polishing and obtain smoother surfaces, being possible to use it for both anterior and posterior restorations, and are called universal composites [15, 24]. In addition, it is of utmost importance that the restorative materials allow a surface smoothness close to that of dental enamel, and this depends directly on the material used [25, 26].

The finishing-polishing procedure of the restorations must be carried out effectively because in this way, it is possible to reduce surface roughness, correct inadequate margins, delimit shape and contour, to give shine and texture to restorations, and highlighting the optical properties of the material [27]. In addition, the correct polishing technique avoids the accumulation of biofilm and gingival irritation, minimizing the risk of caries recurrence and staining, and promoting a greater longevity to the restoration [28, 29].

Surface smoothness acts not only on aesthetic characteristics but also on its durability, since roughness increases the difficulty of cleaning, causing staining and eventual decrease in mechanical properties [30]. Different protocols and materials can be used for finishing and polishing, such as the use of diamond tips, rubber tips, and sanding discs and strips [29, 31]. Surface sealants, which consist of a fluid resin, can also be used to fill microstructural defects to improve mechanical properties and favor staining resistance [31, 32].

The interaction between foods, beverages, and saliva can lead to degradation of properties of restorative materials in the oral cavity [33]. Color change is one of the main factors leading to the replacement of composite resins restorations [34] and may occur in an intrinsic and/or extrinsic way. Factors such as insufficient polymerization of the material and low degree of conversion of the monomers, water absorption, and pigments from food and beverages ingested by the patient can interfere with the color [35]. The microhardness of composite resins can also be affected by the same factors mentioned above, being a property related to the compressive strength and wear resistance of the material [36]. This way, all these factors may directly interfere with the longevity of composite resin restorations in the oral environment.

This study aimed to evaluate the degree of smoothness surface (Ra) provided by two different polishing techniques and the effect of acid challenge on the change of roughness surface, microhardness, and color of three universal nanoparticulate composites, after a 1-year simulation of exposure to hydrochloric acid. The hypotheses tested in this study were that there would be no difference in the degree of surface smoothness provided by two different polishing techniques in any of the nanoparticulate universal composites used, and the different challenges (in artificial saliva or in hydrochloric acid) would not promote changes in the surface, microhardness, and color of any of the composites analyzed.

## 2. Materials and Methods

### 2.1. Study Design and Division of Groups

The factors analyzed in this study were three universal composites, being two of the same commercial brands with different costs between them (Estelite Omega/Tokuyama and Palfique/Tokuyama) and another one from 3M ESPE (Filtek™ Z350 XT, Table 1), four levels of polishing (without polish; wear group with diamond tip; wear group + polish and wear group + surface sealant application), and time at two levels (before and after the acid challenge). The specimens were subjected to surface roughness (Ra—*µ*m), Knoop hardness (KHN, Kgf/mm^2^), and color change (*Δ*E_00_) analysis, before and after challenge acid. The sample size was calculated based on the estimated effect size between groups according to the literature for contact profilometry [20, 37]. It was determined that 10 specimens were needed for each group to achieve a medium effect size (*d* = 0.50), with 0.05 significance and sample size at 80% power level.

Eighty specimens of each of the composites were divided into four groups (*n* = 20), according to the polishing protocol received (Table 2): control without polishing (CWP)—positive control (without polish); control with wear (CW)—negative control (wear performing simulating adjustment with a conical diamond tip, FG 3195 FF, KG Sorensen); WPC—wear + polish with Cosmedent Kit, following this polishing sequence: medium-grained siliconized cups (blue color), extra-fine grain (pink color), and spiral-shaped diamond wheel for high brilliance, diameter 1/2" (pink color), and and WPB—wear + surface sealant applicattion BisCover LV.

Before wear and polishing, an initial roughness reading (Ra) of the surface was taken. In CW, the specimens received about 0.3 mm of wear on one side simulating a finishing with a conical diamond tip 3195 FF, with air/water cooling, for 10 s, and this wear was performed by the same operator on all specimens [38]. For standardization, after wear with a conical diamond tip, the specimens from group WPC were polished for 20 s with each polisher by a single operator. After these procedures, all specimens were subjected to an ultrasonic bath in distilled water for 3 min and air-dried. The surface of the specimens from group WPB, after finishing the wear, was conditioned with 37% phosphoric acid (Condac, FGM) for 15 s, washed for the same time, and dried with air jets. Next, the surface sealant BisCover LV was applied with a disposable microapplicator and polymerized after 15 s of its application using a light curing device (Bluephase; Ivoclar Vivadent) for 30 s, as recommended by the manufacturer.

### 2.2. Obtaining the Specimens for the Surface Roughness, Knoop Microhardness, and Color Change Tests

**Figure 1:**
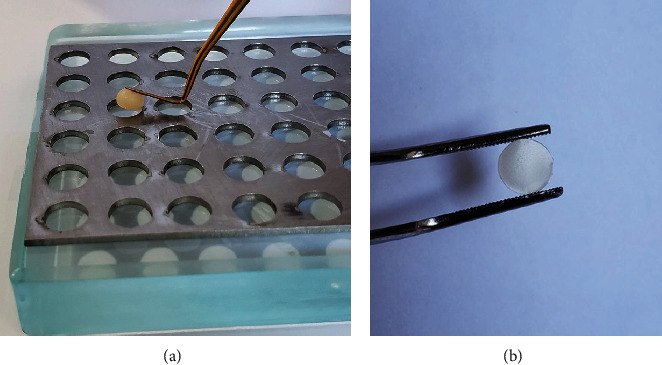
(a) Metal matrix with circular holes was used to make the specimens. (b) Composite resin specimen.

Eighty specimens were made of each of the composites, shown in Table 1, manipulated according to the manufacturers' instructions, totaling 240 specimens. A metal matrix with circular holes was used to make the specimens (Figure 1(a), 6 mm diameter × 1.5 mm thick). The matrix was positioned on a glass plate with a transparent polyethylene strip between them and filled with the composite in a single increment, and then another glass plate was superimposed with another strip of transparent polyethylene, to make it possible to obtain flat and smooth surfaces, and the extravasation of excesses, followed by polymerization for 20 s on one of the specimen surfaces using a photopolymerizer (Bluephase; Ivoclar Vivadent), with a light intensity of 1,200 mW/cm^2^. The glass plates were then removed, and a new direct polymerization was performed for another 20 s on each surface.

The resin excess around the specimen was removed with a scalpel blade. Each specimen received a subtle marking with carbide bur (FG 1/2, KG Sorensen) on the opposite surface so that it was possible to identify the side to be analyzed. These specimens were kept in an oven at 37°C for 24 hr in distilled water (T0) for complete polymerization of the composites. If necessary, to standardize the thickness of 1.5 mm, a metallographic sandpaper with 600 granulation (3M ESPE) was used in a semiautomatic universal polisher (Arotec S.A. Ind Com), with the help of a digital pachymeter (Mitutoyo Sul Americana Ltda.). Finally, the specimens were subjected to ultrasonic cleaning (Ultracleaner 1400; UNIQUE) in distilled water to remove possible debris from the resin surface (Figure 1(b)).

### 2.3. Evaluation of the Surface Roughness of Materials

The measurement of the surface roughness of the composite specimen was performed in a roughness meter (Surftest SJ-400, Mitutoyo Corp., Kawasaki, Japan). The composites specimen were individually positioned in the roughness meter with the surface subjected to analysis always facing upwards, and three parallel readings were taken to measure the initial average surface roughness (Ra—*μ*m) initial (T0), after wear and polish (T1) and final, after acid challenge or immersion in artificial saliva (T2), within a measuring range of 800 *µ*m, with a reading extension of 1.25 mm, with reading intervals of 0.25 mm each (cut-off). The reading speed was 0.1 mm/s. The roughness value was obtained by arithmetic mean and given in micrometers [39].

### 2.4. Evaluation of Microhardness

The microhardness was evaluated using a microhardness tester (Shimadzu HMV-2000; Shimadzu Corporation), equipped with a Knoop diamond, at a load of 25 g for 5 s [40]. A single operator measured the longest diagonal of each marking, and the average of the three indentations with a distance of 100 *μ*m between them, was defined as the average microhardness value (KHN, Kgf/mm^2^) of the specimen before (T1) and after immersion in acid and artificial saliva (T2).

### 2.5. Color Stability Analysis

The specimens were submitted to the initial color reading tests (T1) (baseline—B), using visible ultraviolet reflectance spectrophotometry (UV-2450, Shimadzu, Japan) and after the 91-hr immersion period in artificial saliva or hydrochloric acid (T2), which clinically simulated 1 year of acid exposure [4]. Readings were performed using the CIE L ^*∗*^a ^*∗*^b ^*∗*^ (Commission Internationale de I'Eclairage) color scale, using a D65 illuminant at a 2° observation angle with a wavelength range of 380–780 nm and a 10 nm diameter aperture. Readings were taken by positioning the specimens against a black matte surface [41]. From the central region of the specimen and from only one side of the specimen, the parameters *L* ^*∗*^, *a* ^*∗*^, and *b* ^*∗*^ were obtained and applied to the CIEDE 2000 colorimetric difference formula (*Δ*E_00_), where *L* ^*∗*^ stands for luminosity, *a* ^*∗*^ represents red–green chromaticity, and *b* ^*∗*^ represents yellow–blue chromaticity [42]:

(1) $$\begin{array}{c} \Delta {\text{E}}_{00}={\left[{\left(\frac{\Delta {\text{L}}^{\prime }}{{\text{K}}_{\text{L}}{\text{S}}_{\text{L}}}\right)}^{2}+{\left(\frac{\Delta {\text{C}}^{\prime }}{{\text{K}}_{\text{C}}{\text{S}}_{\text{C}}}\right)}^{2}+{\left(\frac{\Delta {\text{H}}^{\prime }}{{\text{K}}_{\text{H}}{\text{S}}_{\text{H}}}\right)}^{2}+{\text{R}}_{\text{T}}\left(\frac{\Delta {\text{C}}^{\prime }}{{\text{K}}_{\text{C}}{\text{S}}_{\text{C}}}\right)\left(\frac{\Delta {\text{H}}^{\prime }}{{\text{K}}_{\text{H}}{\text{S}}_{\text{H}}}\right)\right]}^{\frac{1}{2}}. \end{array}$$

The *ΔL*, *ΔC*, and *ΔH* are the differences in luminosity (L), chroma (C) and hue (H), respectively, while K is the parametric display factors, and S is the pass functions. The higher the value of *Δ*E_00_, the greater the color change of the material [43]. New readings were taken after the acid challenge. *Δ*E_00_ = 1.30 was considered as the limit of perceptibility and *Δ*E_00_ = 2.25, of clinical acceptability [44].

### 2.6. Acid Challenge Simulation

After the initial readings, the specimens (*n* = 20) were divided into two subgroups (*n* = 10), according to the immersion solution. In the group that was immersed in hydrochloric acid, the specimens were immersed in 0.7 ml of hydrochloric acid (HCl 5%) at pH 2 for 91 hr, which clinically simulated 1 year of acid exposure [4]. In the group that was immersed in artificial saliva, the specimens were immersed in 0.7 ml of artificial saliva, also for 91 hr. During all the time of immersion in both acid and saliva, the specimens were kept in a digital bacteriological oven (CIENLAB Equipamentos Científicos Ltda., Campinas, São Paulo, Brazil) at 37 ± 1°C. When removed from the immersions, the specimens were washed in distilled water three times and dried with absorbent paper. After this period, new readings were taken in order to verify whether the acid challenge contributed to the change in color, roughness, and microhardness of the materials analyzed.

### 2.7. Data Analysis

The surface roughness (Ra), Knoop microhardness, and color change data were subjected to the normal curve adherence test in order to determine whether or not they came from a normal distribution. Since the data showed a normal distribution, repeated measures ANOVA was applied for Ra and Knoop microhardness. For the color change data (*Δ*E), a three-factor ANOVA (Resin × Polishing × Acid Challenge) was applied. In cases of statistically significant difference between the factors analyzed, Tukey's posttest was applied (*α* = 0.05), using the JAMOVI 2.2.5 program.

## 3. Results

### 3.1. Roughness Change Analysis

According to ANOVA, repeated measures of the composite resin specimens, submitted to different polishes and acid challenge, there was no statistically significant difference when the resins were compared among themselves, regardless of time, polish, or acid challenge (*p* = 0.290). In the analysis of variance (ANOVA) repeated measures of each resin, separately, they denote that there was a significant difference in the interaction between the factors Time × Polishing × Challenge for the Z350 (*p*  < 0.001) and Palfique (*p* = 0.010) composite resins, while for the Estelite resin, there was interaction only between the factors Time × Polishing (*p*  < 0.001).

For surface roughness data (Ra—*μ*m), it was possible to observe that there was a significant statistically difference only between the wear group (higher roughness values) when compared to the others, for all composite resin tested (*p*  < 0.05). For the Z350 resin, there was a statistical difference in the roughness of the wear group specimens submitted to HCl immersion (2.40 *μ*m) when compared to specimens immersed in artificial saliva (1.65 *μ*m), as noted in Table 3. However, it was possible to observe that the acid challenge did not promote significant changes on the surface of the specimens between T1 and T2. In the Palfique resin wear group (Table 4), there was a significant increase in surface roughness after acid challenge (2.11 *μ*m). For Estelite resin (Table 5), it was observed that the challenges did not interfere in a statistically significant way.

### 3.2. Analysis of the Change in Knoop Microhardness

There was a significant difference between the resins, regardless of brand (*p*  < 0.001). It was also possible to observe the interaction between the factors Time x Challenge (*p*  < 0.001), Time × Polishing (*p* = 0.019), and Time × Resin (*p*  < 0.001). Among the composite resins analyzed, was observed in Table 6 that the one with the highest microhardness was Z350 (69.3 Kgf/mm^2^), with a statistically significant difference. After immersion in hydrochloric acid and artificial saliva, there was a statistically significant difference among all resins, which had their microhardness decreased. Even after the challenges, the Z350 resin continued to show the highest microhardness value (58.9 Kgf/mm^2^) when compared to the other composites. For all polishing groups, regardless the composite resin brand, there was a significant difference after the immersions, as noted in Table 7, with reduction of microhardness values, regardless of whether the challenge was in artificial saliva or in hydrochloric acid. Regardless of the type of challenge, whether in artificial saliva or in HCL, there was a statistically significant decrease in microhardness values. As observed in Table 8, the specimens immersed in HCL showed lower microhardness (42.4 Kgf/mm^2^) when compared to the specimens immersed in artificial saliva (44.7 Kgf/mm^2^).

### 3.3. Color Change Analysis

It denotes that there was interaction between the factors Resin x Polishing (*p*  < 0.001). Evaluating the color change values (*Δ*E_00_) in Table 9, there was no statistically significant difference between the different polishes for the Estelite and Palfique resins. However, there was a statistically significant difference between the control group and the other types of polishing for the Z350 resin. The highest value of color change was in the control group for the Z350 resin (3.78).

## 4. Discussion

Based on the results obtained, the hypothesis of this study that there would be no difference in the degree of smoothness provided by the polishing techniques employed, for any of the resin brands used, was accepted, as there was no difference between mechanical polishing and chemical polishing, both acted in a similar way in promoting smoothness and maintaining it after the challenges. The polishing and surface smoothness of restorative materials and their chemical composition are factors that influence microbial colonization. Among the required properties of these materials, those related to the surface, such as roughness, are of great clinical importance, as they facilitate biofilm accumulation and staining of the material [45]. As observed in the results of this study, there was no statistically significant difference in the final roughness values of the specimens when immersed in solutions of artificial saliva or hydrochloric acid, showing that the surfaces of these composites were not affected. This fact is very important, since composites, in oral environment, are daily submitted to different pH variations. In this study, specimens were immersed in HCl 5% (pH 2) for 91 hr, representing 1 year of exposure. This time was calculated on the assumption that a bulimic patient purges three times a day for an average of 5 min per purge. Therefore, on average, teeth would be exposed to gastric acid for 15 min a day [46]. One hypothesis that could justify this result is that the exclusive exposure to the acid was not sufficient to alter the surface roughness of the polymers. A study showed that acid alone without the association of brushing abrasion did not change the roughness of the composites studied. The authors observed that toothbrushing for 1 year increased the surface roughness of all the samples when compared to the samples that were exclusively finished and polished or submersed in hydrochloric acid [47].

In the study by Rizzante et al. [32], the BisCover surface sealant showed the lowest surface roughness (Ra < 0.05 *μ*m), a value very close to those obtained in this study, for all composites. Zhang et al. [48] obtained values around 0.27 *µ*m for Z350 XT resin after polishing with aluminum oxide discs; however, this type of polisher is restricted to flatter surfaces, with limitations in its use in occlusal and anterior palatal or lingual areas, so the rubber tips are an excellent option to achieve smoothness in these regions [49]. Compared to the results of the present study, we observed much lower average roughness values for Z350 XT with the Cosmedent Kit (0.09–0.12 *µ*m). Also, according to Zhang et al. [48], the lowest roughness values were obtained by the polyester strips (0.087 *µ*m). In the present study, both types of polishing tested achieved roughness values very close to the values obtained by the control group, demonstrating that the suggested polishing techniques were effective in providing good surface polishing.

Studies establish roughness values (Ra) close to or lower than 0.2 *μ*m to hinder microbial adhesion [50]. In this study, we observed a mean roughness level in the range of 0.06–0.15 *μ*m for the specimens of the composites analyzed, regardless of the commercial brand, and whether analyzed before or after the challenges. This value is below the critical value established by the literature (Ra ≥ 0.2 *μ*m), even after immersion, regardless of if in artificial saliva or in hydrochloric acid. Restorative materials should remain with a smooth surface to avoid biofilm accumulation, staining, surface degradation, and periodontal problems [51].

The hypothesis that different challenges (in artificial saliva or hydrochloric acid) would not promote changes in the surface, microhardness, and color of any of the composites analyzed was partially accepted, since for microhardness and color, they did promote changes. Regarding Knoop microhardness, it was possible to verify that there was a statistically significant difference after the challenges, observing a reduction in the microhardness of the composite specimens, regardless of the commercial brand. According to the literature, it is possible that this reduction in microhardness values is associated with the diffusion of the aqueous medium of these solutions through the resin matrix, affecting the polymer strength and reducing the forces between the polymer chains, causing a change in the mechanical properties of the material [52]. Aqueous solutions were absorbed and act as plasticizers, suggesting a possible change in the chemical structure of composite resins when intensively exposed in these solutions. Surface microhardness provides information about their wear resistance [53]. In de Paula et al.'s [54] study, exposure to any storage solution also produced statistically lower hardness values for all materials tested.

The Knoop microhardness of most composite resins is low if compared to that of dental enamel (343 Kgf/mm^2^). In general, the microhybrid composite resins have Knoop hardness values around 55–80 Kgf/mm^2^, while the microparticulate ones have a much lower average, in a range of 23–36 Kgf/mm^2^, showing that this property is related to the type and volume of filler particles present in each material, meaning that there is a tendency that the resistance to penetration is higher in materials with a higher volume of inorganic filler [55]. In this study, the Knoop microhardness of the resins ranged from 40.9 to 69.3 Kgf/mm^2^ before the challenges and from 34.3 to 58.9 Kgf/mm^2^ after the challenges, showing that the Z350 XT composite resin behaved more compatible with the values of microhybrid composites, and the Palfique and Estelite resins, more compatible with microparticulate composites, although, according to the manufacturers, they all have similar volumes in percentage of inorganic filler. Estelite and Palfique resins have a silica–zirconia filler, with spherical morphology and an average inorganic particle size of 200 nm, and the Z350 resin has a combination of silica particles with 20 nm and Zirconia with 4–11 nm. In other words, the Z350 resin has particles 100 times smaller than the others. Gouveia et al. [56] found values of 106.17 Kgf/mm^2^ in nanocomposite resin samples (Filtek Z350 XT®, 3M/ESPE), observed in the group without treatment and without aging, demonstrating the excellent behavior of this material.

Color change values were evaluated using the *Δ*E_00_ formula, which is based on CIELab coordinates. As a parameter, the perceptibility limit was set at *Δ*E_00_ ≤ 1.30, and the clinical acceptability limit of *Δ*E_00_ ≤ 2.25 [43]. This way, it is observed that the results of *Δ*E_00_ obtained for the tested composites were within the clinical acceptability, presenting values below 2.25, except for the Z350 XT resin, in the control group. Similar results were obtained by Aydin et al. [20] and Patel et al. [57], where the greatest color change observed was in the group in which the specimen surfaces were left in contact with the polyester strip, without receiving any finishing and polishing. Although this strip provides optimal smoothness and inhibits the oxygen layer, the surface obtained presents a rich amount of resin matrix [25], more susceptible to color change, a fact that may have contributed to the higher color change in this group, for this resin. Furthermore, no statistically significant difference was observed between the types of polishings performed, regardless of the resin brand.

These findings are extremely important since intraoral conditions can affect the long-term mechanical and optical properties of materials. Color stability is essential to maintain esthetics over time and to achieve successful and long-lasting restorations. The color alteration of esthetic restorative materials, such as composite resins, is multifactorial, comprising intrinsic and extrinsic factors (staining by food, beverages, cigarettes, among others). The intrinsic factors are related to internal alterations of the material, in its resin matrix, or even the oxidation of residual monomers and tertiary amines [58].

These observations of the present study are particularly important due to the fact that patients ingest acidic beverages and foods on a daily basis, which could potentially cause changes in the properties of restorative materials in general, affecting their longevity, regardless of the commercial brand and price of the product [59]. Methodological limitations are inherent to all in vitro studies. In the current study, resin specimens were immersed in HCl for 91 hr. This was done to simulate the effects of oral cavity exposure to gastric acid for 15 min a day within a short period (1 year) to predict the clinical performance of dental composite materials in terms of surface roughness, microhardness, and color change. Additionally, the presence of water and saliva, occlusal loading, temperature differences, abrasive effects of food, toothbrush and dentifrice, and the pH level in the oral environment can also affect the properties of dental composite restorations, acting together. Finally, although the finishing and polishing procedures were carried out by the same operator, we can not be sure that exactly the same amount of pressure was applied during instrumentations.

## 5. Conclusion

According to the results obtained, it was concluded that both mechanical and chemical polishing resulted in a very satisfactory surface smoothness, even after immersions in artificial saliva and HCl, the microhardness of the composites was affected by challenges in artificial saliva, and HCl and the composites tested were within clinical acceptability with regard to color change.

## Figures and Tables

**Table 1:** Identification of composite resins and surface sealants with regard to their classification and chemical composition.

Material/manufacturer/color	Classification	Chemical composition
Estelite omega/Tokuyama, Japan/EB1	Supranano composite	Bis-GMA (bisphenol A-glycidyl methacrylate) and TEGDMA (triethylene glycol dimethacrylate) monomers; silica-zirconia filler, with spherical morphology and average inorganic particle size of 200 nm (82% by weight or 71% by volume); initiators; stabilizers and pigments

Palfique/Tokuyama, Japan/A2 Esmalte	Supranano composite	Bis-GMA and TEGDMA monomers; silica-zirconia filler, with spherical morphology and an average inorganic particle size of 200 nm (82% by weight or 71% by volume); initiators; stabilizers and pigments

Filtek™ Z350 XT/3M ESPE, USA/B2E	Nanoparticulate composite	Bis-GMA, UDMA (urethanes dimethacrylate), TEGDMA, and Bis-EMA (bisphenol hydroxyethyl methacrylate). Silica filler with size 20 nm nonagglomerated/nonaggregated, zirconia with size 4–11 nm nonagglomerated/nonaggregated and agglomerated, clusters of aggregated zirconia/silica particles (combination of silica particles with 20 nm and Zirconia with 4–11 nm). The inorganic particle loading represents about 78.5% by weight (63.3% by volume)

BisCover LV/BISCO	Low-viscosity surface sealant (liquid polishing)	Dipentaerythritol penta acrylate, ethanol

**Table 2:** Identification of groups, polishing protocols, and challenges.

Groups (for each composite) *n* = 240	Polishing protocols	Challenges (subgroups)
CWP Positive control	Without polish—Specimens were not subjected to finishing/polishing procedures after light-curing under polyethylene strip *n* = 20	Artificial saliva *n* = 10 or HCl 5% *n* = 10
CW Negative control	With wear—The top surfaces of the specimens were ground with a conical diamond tip 3195 FF, with air/water cooling, for 10 s, obtaining 0.3 mm of wear *n* = 20 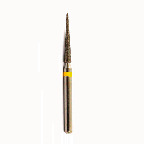	
WPC Wear + polish with Cosmedent Kit	Wear with FG 3195 FF + polishing with medium-grained siliconized cups (blue color), extra-fine grain (pink color), and spiral-shaped diamond wheel for high brilliance, diameter 1/2" (pink color) *n* = 20 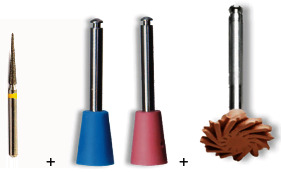	
WPB Wear + surface sealant applicattion BisCover LV	Wear with FG 3195 FF + sealant BisCover LV *n* = 20 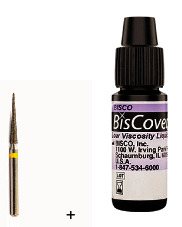	

**Table 3:** Mean values (standard deviation) of roughness (Ra—*µ*m) of Z350 XT composite resins specimens, according to polishing and challenge types.

Groups	Challenges	Initial	After polishing	After challenges
		T0	T1	T2
Control	Saliva	0.07 (0.03) Aa	0.07 (0.03) Aa	0.07 (0.02) Aa
	HCl	0.06 (0.01) Aa	0.06 (0.01) Aa	0.08 (0.01) Aa

Wear	Saliva	0.07 (0.02) Aa	2.02 (0.58) Bb	1.65 (0.43) Bb
	HCl	0.07 (0.02) Aa	2.15 (0.50) Bb	2.40 (0.66) Cb

Biscover	Saliva	0.08 (0.01) Aa	0.08 (0.03) Aa	0.10 (0.05) Aa
	HCl	0.08 (0.02) Aa	0.07 (0.02) Aa	0.09 (0.02) Aa

Cosmedent	Saliva	0.07 (0.02) Aa	0.09 (0.04) Aa	0.08 (0.02) Aa
	HCl	0.07 (0.01) Aa	0.12 (0.02) Aa	0.11 (0.01) Aa

*Note*: Means followed by the same capital letter in the column and lowercase in the row do not differ at 5% significance level (*p* ≤ 0.05) by Tukey's test.

**Table 4:** Mean values (standard deviation) of roughness (Ra—*µ*m) of Palfique LX5 composite resins specimens, according to polishing and challenge types.

Groups	Challenges	Initial	After polishing	After challenges
		T0	T1	T2
Control	Saliva	0.06 (0.01) Aa	0.06 (0.01) Aa	0.07 (0.01) Aa
	HCl	0.06 (0.01) Aa	0.06 (0.01) Aa	0.11 (0.03) Aa

Wear	Saliva	0.07 (0.01) Aa	2.15 (0.68) Bb	1.94 (0.76) Bb
	HCl	0.08 (0.02) Aa	1.66 (0.58) Bb	2.11 (0.42) Bc

Biscover	Saliva	0.07 (0.01) Aa	0.08 (0.03) Aa	0.09 (0.03) Aa
	HCl	0.07 (0.02) Aa	0.08 (0.03) Aa	0.11 (0.04) Aa

Cosmedent	Saliva	0.07 (0.01) Aa	0.11 (0.03) Aa	0.09 (0.02) Aa
	HCl	0.07 (0.02) Aa	0.10 (0.02) Aa	0.15 (0.05) Aa

*Note*: Means followed by the same capital letter in the column and lowercase in the row do not differ at 5% significance level (*p* ≤ 0.05) by Tukey's test.

**Table 5:** Mean values (standard deviation) of roughness (Ra—*µ*m) of Estelite Omega composite resins specimens, according to polishing and challenge types.

Groups	Challenges	Initial	After polishing	After challenges
		T0	T1	T2
Control	Saliva	0.07 (0.01) Aa	0.07 (0.01) Aa	0.08 (0.01) Aa
	HCl	0.07 (0.01) Aa	0.07 (0.01) Aa	0.11 (0.03) Aa

Wear	Saliva	0.07 (0.01) Aa	2.10 (0.71) Bb	2.08 (0.78) Bb
	HCl	0.07 (0.01) Aa	1.97 (0.90) Bb	2.72 (0.89) Bb

Biscover	Saliva	0.07 (0.01) Aa	0.09 (0.03) Aa	0.14 (0.09) Aa
	HCl	0.08 (0.02) Aa	0.08 (0.03) Aa	0.11 (0.04) Aa

Cosmedent	Saliva	0.07 (0.01) Aa	0.10 (0.03) Aa	0.09 (0.02) Aa
	HCl	0.07 (0.02) Aa	0.09 (0.02) Aa	0.13 (0.05) Aa

*Note*: Means followed by the same capital letter in the column and lowercase in the row do not differ at 5% significance level (*p* ≤ 0.05) by Tukey's test.

**Table 6:** Mean values (standard deviation) of Knoop microhardness of the composite resin's specimens according to the brands at different times.

Groups	After polishing	After challenges
	T1	T2
Estelite	40.9 (3.68) Aa	34.3 (4.34) Ab
Palfique	42.5 (5.53) Aa	37.5 (4.32) Bb
Z350	69.3 (7.82) Ba	58.9 (6.07) Cb

*Note*: Means followed by the same capital letter in the column and lowercase in the row do not differ at 5% significance level (*p* ≤ 0.05) by Tukey's test.

**Table 7:** Mean values (standard deviation) of Knoop microhardness of the composite resin's specimens according to the groups after the challenges.

Groups	After polishing	After polishing
	T1	T2
Control	50.9 (12.3) Aa	43.7 (10.4) Ab
Wear	49.1(14.1) Aa	43.1 (11.9) Ab
Biscover	51.4 (15.3) Aa	42.6 (12.0) Ab
Cosmedent	52.2 (15.4) Aa	44.8 (13.9) Ab

*Note*: Means followed by the same capital letter in the column and lowercase in the row do not differ at 5% significance level (*p* ≤ 0.05) by Tukey's test.

**Table 8:** Mean values (standard deviation) of Knoop microhardness of the composite resin's specimens according to the type of challenge.

Groups	After polishing	After challenge
	T1	T2
Saliva	50.5 (14.1) Aa	44.7 (11.8) Ab
Acid (HCl)	51.4 (14.6) Aa	42.4 (12.3) Bb

*Note*: Means followed by the same capital letter in the column and lowercase in the row do not differ at 5% significance level (*p* ≤ 0.05) by Tukey's test.

**Table 9:** Mean values (standard deviation) to color change (*Δ*E), according to the composite resin`s brands after the challenges.

	Estelite	Palfique	Z350
Control	0.756 (0.136) Aa	0.873 (0.335) Aa	3.78 (0.322) Bb
Wear	0.893 (0.283) Aa	0.849 (1.32) Aa	1.33 (1.29) Aa
Biscover	1.41 (0.359) Aa	0.791 (0.318) Aa	1.06 (1.08) Aa
Cosmedent	1.20 (0.451) Aa	1.04 (0.353) Aa	0.834 (0.321) Aa

*Note*: Means followed by the same capital letter in the column and lowercase in the row do not differ at 5% significance level (*p* ≤ 0.05) by Tukey's test.

## Data Availability

The research data used to support the findings of this study have been deposited in the Institucional UNESP Repository (http://hdl.handle.net/11449/236477).
